# Analysis of primary referral patterns and return to work in patients with incident back pain due to lumbar disc herniation

**DOI:** 10.1007/s00701-025-06546-z

**Published:** 2025-05-01

**Authors:** Mikkel Kjeldgaard, Berit Schiøttz-Christensen, Janus Nikolaj Laust Thomsen, Christian Volmar Skovsgaard, Carsten Reidies Bjarkam

**Affiliations:** 1 Department of Neurosurgery, Department of Clinical Medicine, Aalborg University Hospital, 9000 Aalborg, Denmark; 2 Research Unit of General Practice, University of Southern Denmark, Odense, Denmark; 3 Centre for General Practice at, Aalborg University, Aalborg, Denmark; 4 Danish Centre for Health Economics, Department of Public Health, University of Southern Denmark, Odense, Denmark

**Keywords:** Back-pain, Choose wisely, Denmark, Lumbar disc herniation, National registry, Return to work, Work capacity

## Abstract

**Objective:**

To examine primary referral patterns and return to work in patients with incident back pain due to Lumbar Disc Herniation (LDH).

**Methods:**

Nationwide register-based cohort study including all Danish residents aged 18–65 who were referred from primary to specialized healthcare in 2017 with incident back pain and subsequently received a diagnosis of lumbar disc herniation (LDH), defined by ICD-10 codes DM51X.X. Patients were identified using the Danish National Patient Registry (DNPR), including both those directly diagnosed with LDH and those who initially received a diagnosis of nonspecific low back pain (ICD-10: DM54) that progressed to LDH within one year. Demographic data were obtained from the Danish Civil Registration System (CRS), and work capacity outcomes were assessed over a two-year follow-up using the Danish Register for Evaluation of Marginalization (DREAM).

**Results:**

A total of 30,082 persons, corresponding to 0.8% of the Danish population aged 18–65, were referred from primary health care to specialized health care with incident back pain and a final diagnosis of LDH. Of these, 5356 (17.8%) were referred to an emergency department, 14,628 (48.6%) to a medical department, and 10,098 (33.6%) to a surgical department. However, the admission rate and the initial department referred to varied widely between regions. Overall, 1915 (6.4%) underwent surgery. Surgical departments operated more frequently on patients with previous high (11%) or intermediate (14%) work capacity than on those with low work capacity (4%), although the latter were more often referred for surgical evaluation. Over 80% of patients with high or intermediate work capacity maintained or returned to work within a year.

**Conclusion:**

In Denmark, referral from primary to specialized health care of patients with incident back pain due to LDH varies considerable between regions highlighting the need for more standardized referral pathways. Specifically, ensuring a better balance between emergency, medical, and surgical referrals could reduce unnecessary emergency admissions and improve the precision of surgical referrals optimizing the use of surgical capacity and healthcare resources in general.

**Supplementary Information:**

The online version contains supplementary material available at 10.1007/s00701-025-06546-z.

## Introduction

Lumbar Disc Herniation (LDH) is a major cause of low back pain (LBP) and radiculopathy, presenting significant socioeconomic implications due to work absence and reduced productivity [3, 11]. While surgical decompression has demonstrated benefits for carefully selected patients, conservative management remains effective for the majority and several national clinical guidelines (NCG) recommend incidences without severe neurological compromise (red flags) initially to be managed in the primary health care sector [9, 12, 19].

While national databases such as the Danish Spine Database (DaRD) and DaneSpine offer valuable insights into patient-level information on all back disorders across multiple healthcare settings, they do not capture the transition from unspecific back pain to specific diagnoses such as LDH [15]. Previous studies have utilized DaRD data for regional analyses, but its full potential to inform broader clinical practice remains underexploited [14, 15]. Especially, data on the transition from the primary health sector to more specialized spine evaluation and treatment is lacking although such data could provide useful information on current specialized spine health care organization and resource allocation and indicate future directions to optimize patient flow and resource use within our specialties.

Accordingly, the aim of this study is to examine primary referral patterns and their association with return-to-work outcomes in patients aged 18–65 years with incident back pain due to LDH. Using nationwide registry data, we seek to identify regional differences and potential disparities in referral pathways that may influence outcomes and improve clinical decision-making. The focus on incident cases was chosen to ensure that only first-time diagnoses of LDH were analyzed in order to minimize potential bias from previous exposures or treatments and allow for a more accurate assessment of the impact of referral practices on work capacity outcomes.

## Methods

### Data collection

Information on age, gender, and region of residence was obtained from Danish Civil Registration System (CRS). Information on diagnosis (ICD-10 code), initial department at admission, and surgical procedures was accessed through the Danish National Patient Registry (DNPR). The type of department to which patients were initially referred was based on registrations in the DNPR, classifying them into medical, surgical, or emergency departments. Patients were subsequently grouped according to the type of department they were first referred to, allowing for an analysis of referral patterns and treatment outcomes.

Work capacity was assessed using the Danish Register for Evaluation of Marginalization (DREAM), a comprehensive national registry that tracks proportion of registered employment and all public transfer payments on a weekly basis [6, 13]. This measure was derived from the proportion of registered employment, utilizing data from the DREAM [6, 13]. Participants were categorized based on their employment level one year prior to admission into three groups: Low (< 20%), Intermediate (20–80%), and High (≥ 80%) of a standard 37-h work week. Individuals who were unemployed or receiving disability benefits or early retirement pensions were classified as having low work capacity (see limitations for further considerations).

The Danish National Registries contain information of all citizens in Denmark’s five geographical regions, which in 2017 consist of 1.6 million citizens in the Capitol Region, 1.2 million in the Region of Central Denmark and in the Region of Southern Denmark, 812 thousand in the Region of Zealand, and 577 thousand in the Region of Northern Denmark [1]. The year 2017 was chosen to avoid the impact of the COVID19-pandemic, which changed the dynamics of the health system considerably in the 2020–2021 period. The selection of a single year for this study was primarily due to regulatory constraints set forth by the Scientific Ethics Committee in approval of the study. To comply with these regulations and still allow for meaningful follow-up, we focused on one year (2017) to enable both a two-year follow-up period post-diagnosis and a one-year look-back period for prior diagnoses.

### Study population identification

**Fig. 1:**
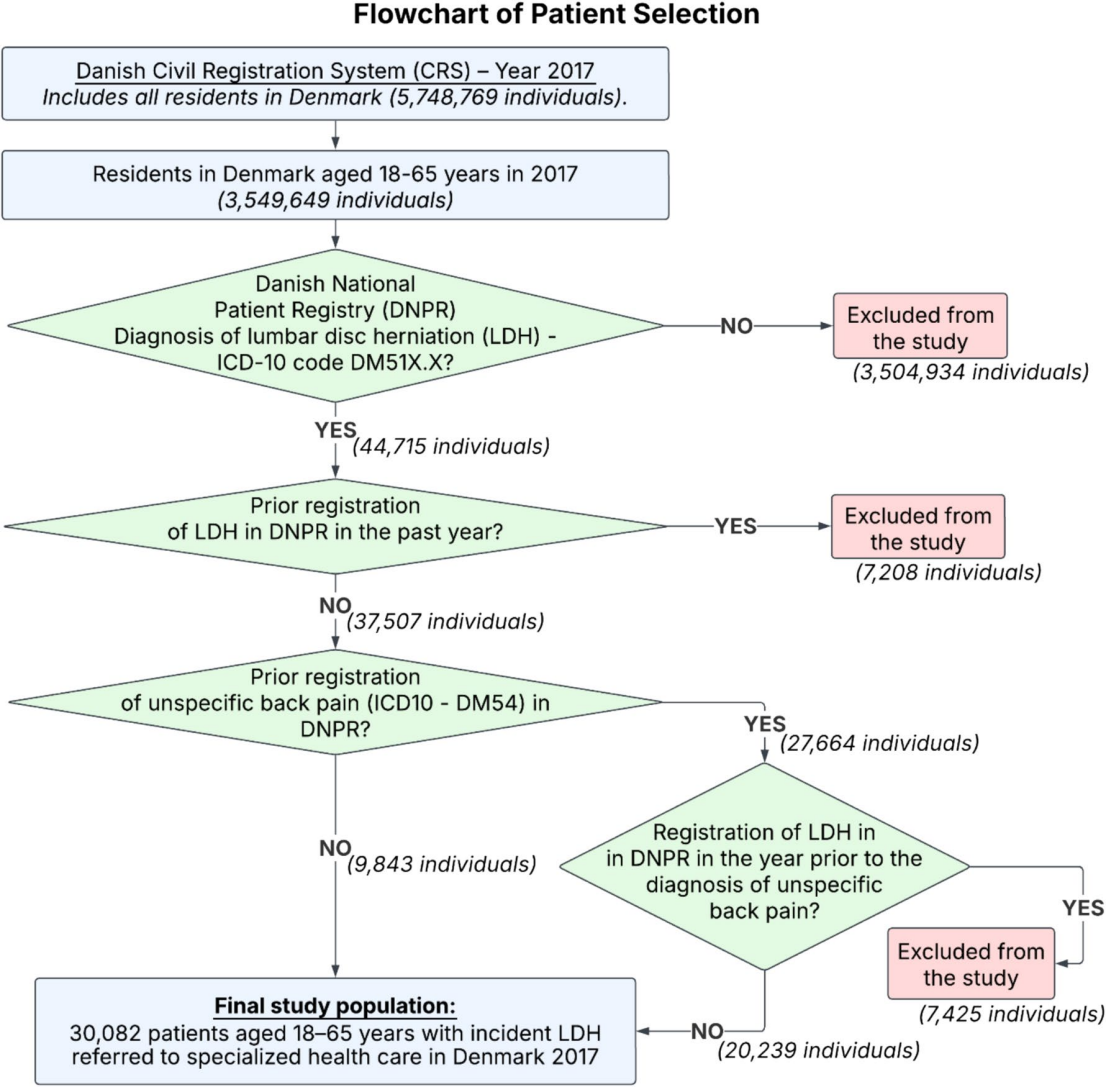
Flowchart of Patient Selection: Using the Civil Registration System (CRS), 3,549,649 individuals aged 18–65 years living in Denmark in 2017 were identified. From the Danish National Patient Registry (DNPR), 44,715 of this cohort were registered with a diagnosis of lumbar disc herniation (LDH) (ICD-10: DM51X.X). Of these, 37,507 had no LDH diagnosis in the previous year. Among them, 27,664 had a prior diagnosis of unspecific back pain (ICD-10: DM54), and 20,239 had no LDH diagnosis within the year preceding their unspecific back pain diagnosis. The final study cohort included 9,843 + 20,239 = 30,082 individuals aged 18–65 years with incident LDH

Incident LDH was defined as a diagnosis of LDH (ICD-10 code DM51X.X) without any prior registration of LDH in the specialized healthcare sector within the preceding year, except for cases initially registered with unspecific low back pain (LBP, ICD-10 code DM54) that subsequently progressed to a specific LDH diagnosis (DM51) within one year. This approach ensured that only patients with first-time diagnoses of LDH were included. Each individual was only counted once. Multiple admissions or diagnoses within the same individual were not considered separate cases. The patient identification process is illustrated in a flowchart found in Fig. 1.

The DM51X.X category covered a range of diagnoses related to lumbar disc herniation, from unspecific (e.g., DM512—Lumbar disc herniation) to more specific diagnoses identifying lumbar disc herniations at a particular level with or without radiculopathy (e.g., DM511.D—L4-L5 disc herniation with radiculopathy) (See Supplementary Material A for full description of subcategory diagnoses). This approach was chosen to ensure a comprehensive analysis of LDH cases, avoiding a substantial reduction in the study population that would have occurred if only the most detailed diagnoses were included. Other spinal pathologies, such as spinal stenosis, were excluded due to their predominance in older populations who are often retired or outside the workforce, the main outcome measure of the study. This exclusion allowed us to focus specifically on referral patterns and outcomes for incident LDH within the working-age population, ensuring that the study's findings generally could be linked to the return-to-work outcomes provided.

In Denmark, patients cannot directly self-refer to surgical units; referrals must be made by a medical doctor, typically through the primary healthcare system. However, some patients may access surgical treatment at private hospitals due to delays in the public healthcare system or through private health insurance. Importantly, data from patients treated at private hospitals are still captured in the DNPR, ensuring their inclusion in this study. This is in accordance with Danish legislation, which mandates that all healthcare providers, both public and private, report patient treatment data to DNPR, regardless of whether the patient is self-referred, referred from public healthcare, self-paying, or covered by insurance [18]. The registry does not provide information to distinguish between public and private healthcare pathways, which represents a limitation of the data.

Inclusion criteria for the study were: (1) individuals aged 18 to 65 years to focus on the working-age population, (2) a diagnosis of LDH recorded in the DNPR (ICD-10 code DM51X.X), (3) no previous diagnosis of LDH in the year prior to the index diagnosis, and (4) patients initially referred with a diagnosis of unspecific low back pain (LBP, ICD-10 code DM54) who, within one year, progressed to a specific diagnosis of LDH.

Exclusion criteria: (1) any prior registration of lower back pain in the specialized healthcare sector within the year preceding the LDH diagnosis, except for cases that progressed from unspecific LBP to LDH within one year, (2) individuals outside the 18–65 age range, (3) and incomplete data for key variables such as age, diagnosis, or referral pathways.

Eligible patients were further stratified based on the DNPR categorization of departments to which they were initially referred: emergency departments, medical departments, and surgical departments.

### Exposure and outcome variables

The exposure variable was defined as the type of healthcare department to which patients with LDH were initially referred—categorized into medical, surgical, or emergency departments. Departments were classified as medical, surgical, or emergency based on their registration in the Danish National Patient Registry (DNPR). The outcome variables were defined as work capacity prognosis and surgical intervention status. The work capacity prognosis was measured as the time taken to regain the same level of employment as one year prior to admission, assessed over a 2-year follow-up period at 6-month intervals. The surgical rate was defined as the proportion of patients who underwent at least one surgical intervention within each identified cohort and was presented per 10,000 patients. This was calculated by dividing the number of individuals who received at least one surgical procedure—identified using SKS codes detailed in Supplementary A—by the total number of patients in the respective cohort. Patients were only counted once, regardless of whether they underwent multiple procedures. Supplementary B details the demographic characteristics and work capacity outcomes among (i) patients with low baseline capacity who improved, and (ii) those who remained below the 20% threshold after two years. Supplementary C compares demographic and work outcomes between surgically and non-surgically treated patients.

### Statistical analysis

Descriptive statistics including frequencies and percentages were used to summarize referral patterns, work capacity outcomes, and surgical rates by region and department type. Results are presented in tables and figures.

All data management of register data was programmed and designed by the study group for this study population using STATA [17].

## Results

The study population was based on all 3,549,649 Danish residents aged 18–65 years in 2017, identified through the Danish Civil Registration System (CRS). Among these, 44,715 individuals received a lumbar disc herniation (LDH) diagnosis (ICD-10: DM51X.X) in specialized healthcare that year, according to the Danish National Patient Registry (DNPR). After applying the study’s eligibility criteria — excluding patients with a previous LDH diagnosis — we identified 9,843 patients who were initially referred with an incident LDH diagnosis. In addition, 20,239 patients were first referred with nonspecific low back pain (ICD-10: DM54) but progressed to a specific LDH diagnosis (DM51X.X) within one year. Combined, the total study cohort included 30,082 patients. The full patient identification process is illustrated in Fig. 1.

**Fig. 2:**
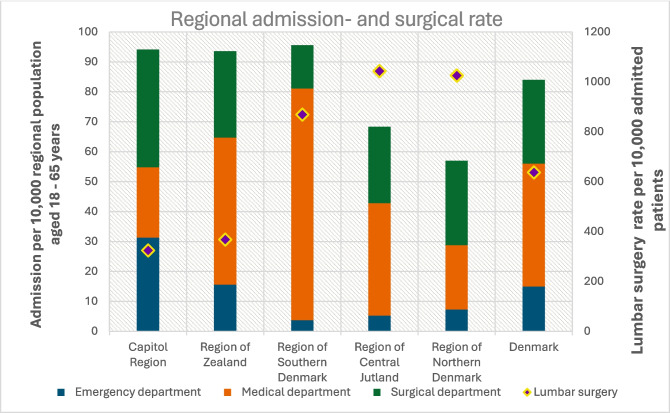
Regional admission and surgical rates. Regional admissions per 10,000 of the regional general population aged 18–65 years//Regional surgery rate per 10,000 admitted patients aged 18–65 years//surgical rates are presented as discrete, region-specific values

Table 1 and Fig. 2 summarize the demographic characteristics of the study population and regional referral patterns across department types at initial admission. The national admission rate was 85 per 10,000 general population aged 18–65 years, however, notable regional differences in admissions were observed, ranging from 57 to 96 per 10,000 of each Region’s individual population aged 18–65 years (Table 1). On a national level, 5,356 patients (17.8%) were initially referred to an emergency department, 14,628 (48.6%) to a medical department, and 10,098 (33.6%) to a surgical department. However, there were marked interregional differences in referral patterns (Table 1 & Fig. 2). For example, the Region of Northern Denmark referred nearly half of its patients (49.5%) to surgical departments, whereas the Region of Southern Denmark referred only 15.1% to surgical care. Conversely, the Capital Region had the highest proportion of emergency referrals (33.3%).

**Table 1:** Regional admission and surgical rates. * Regional admissions per 10,000 of the regional general population aged 18–65 years//** Regional surgery rate per 10,000 of the admitted population aged 18–65 years

						Denmark		
	Capitol Region	Region of Zealand	Region of Southern Denmark	Region of Central Jutland	Region of Northern Denmark	Percentage distribution (%)	Rate (*n* per 10,000)	*Total (n)*
General population aged 18–65 years (*n*) *	1,159,101	494,021	733,449	805,270	357,808			*3,549,649*
Total admission (*n*)	10,909	4,621	7,010	5,503	2,039			*30,082*
Admission per 10,000 of the general regional population aged 18–65 years*	94	94	96	68	57		85	
First department at admission								
Emergency department (intra-regional department distribution (%))	33.3	16.7	3.9	7.7	12.8	17.8		*5,356*
Medical department (intra-regional department distribution (%))	25.0	52.5	81.0	55.0	37.7	48.6		*14,628*
Surgical department (intra-regional department distribution (%))	41.7	30.8	15.1	37.3	49.5	33.6		*10,098*
Lumbar surgery rate per 10,000 admitted population **	324	368	869	1,043	1,025		637	*1,915*
Emergency department (n per 10,000 in department) **	513	2,338	364	2,725	192		624	*334*
Medical department (n per 10,000 in department) **	220	280	680	330	325		437	*639*
Surgical department (n per 10,000 in department) **	235	590	2,013	1,750	1,774		933	*942*
Unspecific back pain diagnosis (ICD-10: DM54) prior to final diagnosis of lumbar disc herniation (%)	71.0	73.4	67.8	55.3	64.1	67.0		*20,241*

*Regional admissions per 10,000 of the regional general population aged 18–65 years

**Regional surgery rate per 10,000 of the admitted population aged 18–65 years

The overall surgical treatment rate was 637 per 10,000 admitted patients (Table 1). Surgical rates varied by department: surgical departments had the highest rate at 933 per 10,000, emergency departments had 624 per 10,000, and medical departments had the lowest at 437 per 10,000 (Table 1 & Fig. 2). The surgical treatment rate varied likewise considerably (Table 1 and Fig. 2), even in regions with similar admission rates. Thus, the Capitol Region had a surgery rate of 324 per 10,000, while the Region of Southern Denmark had 869 per 10,000 (Table 1).

Most of the study cohort, approximately 67% of patients, had a diagnosis of unspecific back pain prior to their subsequent final diagnosis of LDH and their following inclusion in this study. While generally high, the proportions differed likewise across the health care regions of Denmark ranging from 55–73% of the referred patients in this study.

**Fig. 3:**
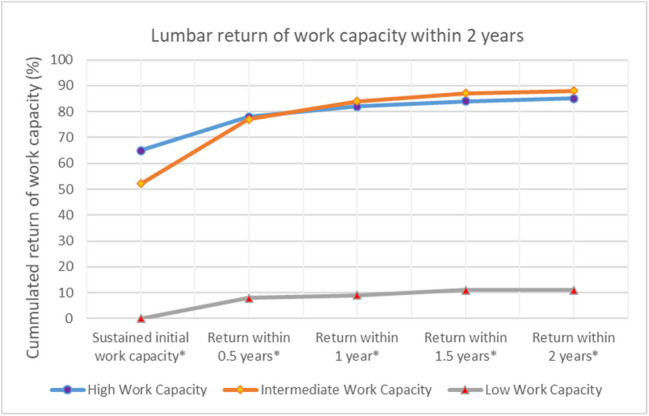
Cumulated return of work capacity within 2 years in percent (%)//HWC: High Work Capacity; IWC: Intermediate Work Capacity; LWC: Low Work Capacity//* Total percentage of patients with return to a work capacity as one year prior to admission OR achieving a work capacity ≥ 20% if previous low work capacity (%)//Subjects with missing information excluded in this analysis

Table 2 and Fig. 3 present the study population stratified by prior work capacity. Among the cohort, 71.1% had high work capacity, 8.5% intermediate, and 21.4% low. Patients with high or intermediate work capacity were most frequently referred to medical departments, while 45% with low work capacity were referred to surgical departments, despite only 4% underwent surgery. In the Capitol Region, 59% of patients with low work capacity were referred to surgical departments—considerably more than the 34–35% seen in those with higher work capacity.

**Table 2:** Descriptive statistics of the study population according to high-, intermediate-, or low work capacity one year prior to admission//ED: Emergency department; MD: Medical department; SD: Surgical department; WC: Work capacity//* National intraregional department distribution in percent (%)//** Total percentage of patients with return to a work capacity as one year prior to admission, or achieving a work capacity ≥ 20% if previous low work capacity (%) *** Subjects with missing information excluded in this analysis

	Work Capacity ≥ 80%					Work Capacity [< 80%; ≥ 20%]					Work Capacity < 20%				
	*High Work Capacity*					*Intermediate Work Capacity*					*Low Work Capacity*				
Department	ED	MD	SD	Total	n	ED	MD	SD	Total	n	ED	MD	SD	Total	n
National intradepartment distribution (%)	20	50	30	100	*21,087*	19	51	30	100	*2,568*	11	44	45	100	*6,427*
Region of the hospital															
Capitol Region (%)*	**39**	25	36	100	*7,063*	**42**	24	34	100	*806*	17	24	**59**	100	*3,040*
Region of Zealand (%)*	18	**52**	30	100	*3,402*	16	**54**	30	100	*410*	13	**56**	31	100	*809*
Region of Southern Denmark (%)*	4	**82**	14	100	*5,225*	5	**78**	17	100	*655*	3	**77**	20	100	*1,130*
Region of Central Jutland (%)*	9	**55**	36	100	*3,798*	7	**59**	34	100	*509*	4	**55**	41	100	*1,196*
Region of Northern Denmark (%)*	13	37	**50**	100	*1,599*	11	46	**43**	100	*188*	15	35	**50**	100	*252*
Surgery rate in percent of admitted patients (%)	6	5	**11**	7	*1,494*	6	3	**14**	7	*182*	**5**	3	4	4	*239*
Return rate to previous WC OR time before gaining a WC ≥ 20% if previous low WC ** & ***															
Sustained initial work capacity (%)**	70	71	72	71	*13,740*	60	54	59	57	*1,338*					
Return within 0.5 years (%)**	15	14	14	14	*2,766*	26	28	28	27	*647*	15	10	5	8	*510*
Return within 1 year (%)**	4	5	4	4	*925*	7	9	7	8	*174*	1	1	1	1	*52*
Return within 1.5 years (%)**	2	2	2	2	*396*	3	3	3	3	*74*	4	3	2	2	*157*
Return within 2 years (%)**	1	1	1	1	*170*	1	1	1	1	≤ *26*	1	1	0	0	*32*
2 years persistent loss of work capacity (%)**	*7*	*5*	*7*	*7*	*1,183*	*3*	*4*	*2*	***3***	*84*	*79*	*85*	*92*	*89*	*5,676*

Despite regional differences in admission and surgical rates, the overall prognosis of return to work remained favorable for patients with a previous high or intermediate work capacity: Specifically, 71% and 57% (Table 2 & Fig. 3) maintained their work capacity throughout the 2-year study period. Within 0.5 years, a further 14% and 27% of the two groups regained their lost work capacity. The likelihood of regaining prior work capacity declined after 0.5 years. In contrast, 89% of patients with a previous low work capacity did not gain any work capacity (> 20% occupational hours of a normal work week). However, 8% showed an increase to a work capacity of > 20% within 0.5 years, with an additional 1% and 2% increase within one and 1.5 years, respectively.

## Discussion

This nationwide study examined referral patterns and return-to-work outcomes in patients with incident lumbar disc herniation (LDH) referred from primary healthcare to specialized health care in Denmark. To be further elaborated below, we identified substantial regional differences in both initial referral departments and surgical intervention rates. Despite these variations, the overall surgical intervention rate among referred patients remained low at 6.4%. Furthermore, return-to-work outcomes were generally favorable, particularly among patients with high or intermediate baseline work capacity.

The national average admission rate was 85 per 10,000, equivalent to 0.85% of citizens aged 18–65 in Denmark (Table 1). Interestingly, the Region of Northern Denmark (57/10,000) and the Region of Central Jutland (74/10,000) had among the *lowest* admission rates nationwide, yet reported some of the *highest* surgical intervention rates, with 869 and 775 surgeries per 10,000 admitted patients, respectively. This contrasts with regions such as the Capitol Region (94/10,000) and the Region of Southern Denmark (96/10,000), which had much higher referral rates but significantly lower surgical rates (324 and 435 per 10,000 admitted patients, respectively). These patterns suggest that the number of surgical candidates may remain relatively stable across regions, and that lower referral rates may lead to a higher surgical conversion rate, potentially reflecting more targeted or appropriate referrals.

Although most regions initially refer patients to medical spine centers, substantial variations exist in the initial referral departments. In Northern Denmark, nearly half (49.5%) of patients were initially referred to surgical departments, compared to only 15.1% in the Region of Southern Denmark and a national average of 33.6% (Table 1). This may be attributed to differences in the classification of healthcare facilities in national registries, such as the Middelfart spine center being coded solely as a medical facility despite offering combined care. Similarly, the high proportion of emergency referrals in the Capitol Region may reflect structural factors such as the centralized after-hours triage system, which may result in more non-urgent patients being referred to emergency departments (Table 1). While emergency referrals are appropriate for acute presentations, the volume observed appears excessive and may be less cost-effective. Emergency physicians are adept at ruling out serious conditions such as neural compromise or trauma; however, in the absence of such findings, most patients with lumbar disc herniation (LDH) are discharged without definitive intervention. This often necessitates subsequent referral by general practitioners to specialized spine clinics, potentially delaying both diagnosis and appropriate treatment.

Referral patterns also varied according to patients'baseline work capacity. Individuals with low work capacity were more frequently referred to surgical departments, despite exhibiting the lowest rates of surgical intervention. Nationally, while 33.6% of all patients referred to specialized health care in this study were initially referred for surgical evaluation, only 6.4% of the total study cohort ultimately underwent surgery (Table 1). Notably, among patients with low work capacity, 45% were referred directly to surgical departments, yet just 4% received surgery (Table 2). This discrepancy raises concerns regarding the appropriateness and efficiency of surgical referrals.

Our return-to-work data further suggest that incident back pain related to LDH is largely a benign condition despite national disparities in admission and surgical rates. Specifically, 71% of individuals with a high work capacity and 57% with an intermediate work capacity one year prior to admission were able to maintain their work capacity for two years following their initial referral (Table 2). Within the first half-year this percentage further grew to 85% of individuals with high work capacity and 84% with intermediate work, whereafter the recovery rate declined steeply (Table 2). These findings align with previous studies on surgical and conservative treatment regimens of disc herniations [2, 8, 10]. While sustained or return to work capacity does not necessarily exclude the presence of persistent pain and symptoms, it is widely regarded in epidemiological studies as a robust indicator of disease severity [5, 7].

In summary, while we acknowledge that spine surgeons probably are the best qualified to identify appropriate surgical candidates, the low surgical rate per 10,000 patients referred to surgical departments raises concern. It suggests that considerable surgical resources are spent on evaluating patients who ultimately do not undergo surgery, many of whom are likely re-referred to specialized medical spine care. This may result in unnecessary delays in the treatment pathway and inefficient use of outpatient capacity. From the perspective of the'Choose Wisely'paradigm, we recommend that most primary referrals could be initially directed towards specialized medical spine evaluation. Emergency or early surgical department referrals should generally be reserved for patients presenting with severe neurological deficits, such as cauda equina syndrome, progressive symptoms, or intractable pain. Importantly, there should still be room for timely surgical evaluation of patients with incident back pain from LDH, as discectomy has been shown to be an effective treatment for symptomatic LDH facilitating pain relief and a quicker return to work for selected patients [2, 8, 10]. Accordingly, some clinical guidelines recommend surgical evaluation after 6–8 weeks of persistent symptoms if conservative treatment fails to provide relief [19].

### Study strengths and limitations

A key strength of this study is the use of a large, unselected cohort drawn from a nationwide registry, which enhances the external validity of our findings, especially if the results are extrapolated to other countries with a Scandinavian-like gratuitous (tax financed) health-care system and a resulting less dominant private health care sector. However, an inherent limitation lies in the assumption that patients referred with LBP ultimately correspond to the assigned LDH diagnoses. To address this, we employed a structured approach, including the use of diagnostic codes and referral pathways, to identify cases of incident LDH within the broader group of LBP patients. While this approach increases the validity of our findings, the potential for misclassification, e.g. information bias remains, particularly for patients whose conditions were not fully captured in the registry.

All individuals who were unemployed or receiving disability benefits or early retirement pensions were classified as having low work capacity. This categorization may not fully capture variations in work potential and represents a limitation of the study design. The authors believe this limitation is minimal, as Denmark has a relatively low unemployment rate of approximately 2.9% of December 2024, and the proportion of individuals receiving disability benefits or early retirement pension before the age of 65 is also relatively small (< 7.5% of citizens aged 18–65 years in Denmark in 2024) [4, 20].

The referral decisions by general physicians may be influenced by subjective assessments of the severity of symptoms, leading to a non-uniform distribution of patients across departments. For example, patients with similar symptoms might be referred to different departments (e.g., medical vs. surgical) depending on the physician’s clinical judgment, local policies, or regional healthcare protocols. This variation can skew the representation of disease severity in each department, affecting the comparability of patient outcomes across regions. Thus, because of the probable selection bias one should be careful to compare the outcome result (return to work) between departments as most patients hopefully are not randomly referred to the different department types. Accordingly, the data provided are not useful to elucidate if surgery is better or worse than conservative medical treatment but should rather be seen to provide insight into the referral pattern of patients with incident LDH related back pain and their ability to maintain work capacity or return to work.

We acknowledge that a key limitation of registry-based studies is the inability to determine the specific reason for referrals, such as surgical evaluation, diagnostic clarification, or pain management. The DNPR records the type of department patients are referred to but does not capture the underlying clinical rationale for the referral. A limitation of this study is the potential classification bias in department type (Middelfart Rygcenter) and diagnosis (the validity of the given DM51X.X diagnosis). In order to include all patients with LDH we included all patients assigned to a DM51X.X diagnosis. Some diagnoses within this group are quite unspecific (e.g. DM512 Lumbar disc herniation) while others are more specific, identifying lumbar disc herniations at a particular level with radiculopathy (e.g., DM511.D—L4-L5 disc herniation with radiculopathy). Focusing solely on more specific diagnoses would have substantially reduced the study population due to the limited detail available in the registries. By including the broader DM51X.X category, the study aimed to balance diagnostic specificity with population representativeness, ensuring that the analysis remained both comprehensive and relevant. It should however be clearly stated that although most patients referred to specialized health care receive an MR-scan, this is not a prerequisite for a DM51X.X diagnosis. Accordingly, the initial high number of unspecific back pain diagnoses (DM54X.X) in our cohort probably reflects that on the way through the system these patients receive an MRI thereby ultimately receiving a more specific LDH diagnosis.

Although registry-based studies can be limited by potential misclassification of diagnoses, existing validation-studies indicate that the Danish National Patient Registry (DNPR) maintains a high level of diagnostic accuracy. For instance, the positive predictive value (PPV) for primary diagnoses is reported to be 73%, rising to 83% for orthopedic surgery diagnoses [16]. Unlike many registry-based studies that rely on voluntary reporting or regional data, the DNPR captures all hospital, public and private, contacts nationwide, minimizing the risk of selection bias. Additionally, the homogeneity of the Danish healthcare system, which ensures free and equal access to care, reduces the impact of socioeconomic factors on referral patterns and treatment access [16].

## Conclusion

In Denmark, referral from primary to specialized health care of patients with incident back pain due to LDH varies considerable between regions, emphasizing the need for more standardized referral pathways. Specifically, ensuring a better balance between emergency, medical, and surgical referrals could reduce unnecessary emergency admissions and improve the precision of surgical referrals optimizing the use of surgical capacity and healthcare resources in general.

## Supplementary Information

Below is the link to the electronic supplementary material.

Supplementary file1 (PDF 325 KB)

## Data Availability

No datasets were generated or analysed during the current study.

## Abbreviations

CRS: Danish Civil Registration System
DaRD: Danish Spine Database
DaneSpine: Danish National Spine Surgery Registry
DNPR: Danish National Patient Registry
DREAM: Danish Register for Evaluation of Marginalization
ED: Emergency Department
HWC: High Work Capacity
ICD-10: International Classification of Diseases, 10th Revision
IWC: Intermediate Work Capacity
LBP: Low Back Pain
LDH: Lumbar Disc Herniation
LWC: Low Work Capacity
MD: Medical Department
NCG: National Clinical Guideline
SD: Surgical Department
SKS: Health Care Classification System
