# Supplementary material for: Analysis of primary referral patterns and return to work in patients with incident back pain due to lumbar disc herniation
Source: Acta Neurochir (Wien). 2025 May 1;167(1):129. doi: 10.1007/s00701-025-06546-z (PMC12045806; doi:10.1007/s00701-025-06546-z)
Supplement: Supplementary file 1 — Supplementary file1 (PDF 325 KB) [file 701_2025_6546_MOESM1_ESM.pdf]

Supplementary materials

Supplementary A – variable characteristics

Table 1 Variable characteristics

Variables	Type	Subtype	Categories	Codes
**Region of admission**	Categorical	Nominal	Capitol Region;<br>Region of Zealand;<br>Region of Southern Denmark;<br>Region of Central Jutland;<br>Region of Northern Denmark	
**Admission department**	Categorical	Nominal	Emergency-, medical- or surgical department	
**Diagnosis**	Categorical	Nominal	Lumbar disc herniation, back pain of no other specification. (ICD-10 codes)	Lumbar intervertebral disc herniation: DM51<br><br>Unspecified back pain: DM54
**Diagnosis subcategories of DM51 Lumbar disc herniation**<br><br>*This section outlines all ICD-10 subcategories used to define LDH and associated diagnoses included in the study.*	Lumbar intervertebral disc herniation: DM51<br>Lumbar disc disorder with myelopathy: DM51.0<br>Lumbar disc disorder with radiculopathy: DM51.1<br>Other specified intervertebral disc displacement: DM51.2<br>Other intervertebral disc degeneration: DM51.3<br>Schmorl's nodes: DM51.4<br>Other intervertebral disc disorders: DM51.8<br>Intervertebral disc disorder, unspecified: DM51.9<br>Subcategories of DM511 (Radiculopathy):<br>Lumbar disc herniation I/II with radiculopathy: DM511A<br>Lumbar disc herniation II/III with radiculopathy: DM511B<br>Lumbar disc herniation III/IV with radiculopathy: DM511C<br>Lumbar disc herniation IV/V with radiculopathy: DM511D<br>Lumbosacral disc herniation with radiculopathy: DM511E<br>Lumbar disc herniation UNS with radiculopathy: DM511F<br>Thoracic disc herniation with radiculopathy: DM511H<br>Thoracolumbar disc herniation with radiculopathy: DM511I<br>Subcategories of DM512 (Unspecified disc herniation):<br>Lumbar disc herniation I/II UNS: DM512A<br>Lumbar disc herniation II/III UNS: DM512B<br>Lumbar disc herniation III/IV UNS: DM512C<br>Lumbar disc herniation IV/V UNS: DM512D<br>Lumbosacral disc herniation UNS: DM512E<br>Lumbar disc herniation UNS: DM512F<br>Thoracic disc herniation UNS: DM512G<br>Other related diagnoses:<br>Lumbar disc degeneration UNS: DM513A<br>Thoracic disc degeneration UNS: DM513B<br>Disc calcification: DM513C<br>Schmorl's nodes: DM514<br>Other diseases of lumbar or thoracic intervertebral discs: DM518			

				Unspecified disease of lumbar or thoracic intervertebral discs: DM519
**Surgery**	Categorical	Nominal	Lumbar surgery of lumbar disc herniation(-s) (SKS codes)	KABC07, KABC16, KABC26, KABC36, KABC40, KABC56, KABC66, KABC07, KABA00, KABA99
**Work capacity 1 year prior**	Numeric	Continuous	(37hours/week reference)	
**Return time to regain initial work capacity**	Categorical	Ordinal	No change, 0.5, 1, 1.5, 2 years	

Supplementary B – Analysis of no-return and gained no previous work capacity

	No recovery of a previous High Work Capacity (≥80%)				No recovery of a previous Intermediate Work Capacity [<80%;≥20%]				Low Initial Work Capacity (<20%) who Improved Work Capacity (≥20%)			
**Department**	**ED**	**MD**	**SD**	***n***	**ED**	**MD**	**SD**	***n***	**ED**	**MD**	**SD**	***n***
Department distribution (%)*	20	47	33	*1183*	20	59	21	*1183*	20	51	29	*1183*
**Region of the hospital**												
Capitol Region (%)*	38	24	39	*402*	57	22	22	*23*	45	27	28	*220*
Region of Zealand (%)*	18	42	40	*188*	10	70	20	*≤15*	23	45	32	*137*
Region of Southern Denmark (%)*	7	79	14	*290*	0	89	11	*≤29*	3	82	15	*207*
Region of Central Jutland (%)*	10	52	38	*212*	12	47	41	*≤19*	10	55	36	*143*
Region of Northern Jutland (%)*	11	43	46	*91*	0	83	17	*≤9*	8	30	62	*≤50*
**Return to previous work capacity**												
Return within 0.5 years (%)**									70	70	61	*510*
Return within 1 year (%)**									6	7	8	*52*
Return within 1.5 years (%)**									19	18	26	*157*
Return within 2 years (%)**									4	5	4	*32*
2-years persistent loss of work capacity (%)**	100	100	100	*1183*	100	100	100	*≤90*				

* Intradepartmental distribution in percent (%) // ** cummulated return of work capacity in percent (%)

Supplementary C – Analysis of surgical and no-surgical treatment groups according to type of department

	All recieving surgery				No surgery			
**Department**	**ED**	**MD**	**SD**	Total (n)	**ED**	**MD**	**SD**	Total (n)
**Intradepartmental distribution (%)***	8	29	64	*≤1917*	18	50	33	*28167*
**First registered with UNS**	25	29	23	*492*	88	68	63	*19749*
**Return to previous work capacity**								
Sustained full work capacity (%)**	59	55	56	*1072*	54	51	45	*14006*
Return within 0.5 years (%)**	23	14	15	*274*	15	13	11	*3649*
Return within 1 year (%)**	3	6	5	*92*	4	4	3	*1059*
Return within 1.5 years (%)**	2	3	2	*47*	3	2	2	*580*
Return within 2 years (%)**	≤1	1	1	*≤15*	1	1	1	*213*
2-years persistent loss of work capacity (%)**	13	16	14	*285*	16	21	32	*6658*

ED: Emergency department; MD: Medical department; SD: Surgical department // * Intradepartmental distribution in percent (%) // ** Cummulative return of work capacity in percent (%)
